# A defect in the peroxisomal biogenesis in germ cells induces a spermatogenic arrest at the round spermatid stage in mice

**DOI:** 10.1038/s41598-019-45991-6

**Published:** 2019-07-02

**Authors:** Ann-Kristin Brauns, Markus Heine, Klaus Tödter, Eveline Baumgart-Vogt, Georg H. Lüers, Udo Schumacher

**Affiliations:** 1 Department of Anatomy and Experimental Morphology, University Medical Center Hamburg-Eppendorf (UKE), 20246 Hamburg, Germany; 2 Department of Biochemistry and Cell Biology, University Medical Center Hamburg-Eppendorf (UKE), 20246 Hamburg, Germany; 3 Institute of Anatomy and Cell Biology II, Justus Liebig University, 35385 Giessen, Germany

**Keywords:** Peroxisomes, Testis

## Abstract

Peroxisomes are involved in the degradation of very long-chain fatty acids (VLCFAs) by *β*-oxidation. Besides neurological defects, peroxisomal dysfunction can also lead to testicular abnormalities. However, underlying alterations in the testes due to a peroxisomal defect are not well characterized yet. To maintain all metabolic functions, peroxisomes require an import machinery for the transport of matrix proteins. One component of this translocation machinery is PEX13. Its inactivation leads to a peroxisomal biogenesis defect. We have established a germ cell-specific KO of *Pex13* to study the function of peroxisomes during spermatogenesis in mice. Exon 2 of floxed *Pex13* was specifically excised in germ cells prior to meiosis by using a transgenic mouse strain carrying a STRA8 inducible *Cre* recombinase. Germ cell differentiation was interrupted at the round spermatid stage in *Pex13* KO mice with formation of multinucleated giant cells (MNCs) and loss of mature spermatids. Due to a different cellular content in the germinal epithelium of *Pex13* KO testes compared to control, whole testes biopsies were used for the analyses. Thus, differences in lipid composition and gene expression are only shown for whole testicular tissue but cannot be limited to single cells. Gas chromatography revealed an increase of shorter fatty acids and a decrease of n-6 docosapentaenoic acid (C22:5n-6) and n-3 docosahexaenoic acid (C22:6n-3), the main components of sperm plasma membranes. Representative genes of the metabolite transport and peroxisomal *β*-oxidation were strongly down-regulated. In addition, structural components of the blood-testis barrier (BTB) were altered. To conclude, defects in the peroxisomal compartment interfere with normal spermatogenesis.

## Introduction

Peroxisomes are ubiquitous eukaryotic cell organelles that are essential to maintain cellular function and homeostasis. One of their major functions is the degradation of VLCFAs and their derivatives via *β*-oxidation. Even though still under debate, they at least partially contribute to the synthesis of steroids and cholesterol^1^. In addition, they are involved in the biosynthesis of glycerolipids and bile acids, the catabolism of purines and polyamines, as well as the degradation of reactive oxygen species (ROS)^1^.

Peroxisomes are generally formed by growth and division^2^ or are derived *de novo* from the ER^3,4^. Peroxisomal membrane and matrix proteins are translated in the cytosol on free ribosomes or on ER-associated ribosomes and directly targeted to peroxisomes by means of e.g. PEX19 that acts as chaperone for newly synthesized peroxisomal membrane proteins in the cytosol and directs cargo to the peroxisomal membrane and thereby functions as shuttling receptor^5,6^. The formation of the peroxisomal membrane, peroxisome proliferation and compartmentalization of peroxisomal matrix proteins is maintained by peroxins (PEX proteins)^7,8^. All peroxisomal matrix proteins harbor a peroxisomal targeting signal type 1 (PTS1) or type 2 (PTS2) at the C- or N-terminus, respectively^9^. The targeting signals are recognized in the cytosol by the cognate peroxisomal import receptors (e.g. PEX5 and PEX7) that cycle between the cytosol and the peroxisomal membranes^10,11^. The receptor/cargo complex interacts with the peroxisomal import machinery, composed of PEX13 and PEX14 in human. The cargo is translocated over the membrane and released into the peroxisomal matrix, whilst the receptor is recycled^12^. Studies showed a severe impact on the import pathway of cargos through absent PEX13 or PEX14^13,14^. The translocation of substrates for peroxisomal *β*-oxidation is facilitated through the ATP-binding cassette (ABC) transporter of subfamily D (ABCD)^15–17^. Accumulated unbranched, saturated (SFAs) and very long-chain fatty acids in the cell, as a consequence of mutated *Abcd1*, result in myelopathies as described for the X-linked adrenoleukodystrophy (X-ALD) or its milder form, the adrenomyeloneuropathy (AMN)^18–20^. Besides adrenocortical dysfunction, patients also have lesions in their testicular interstitial cells and smaller seminiferous tubules with a spermatogenic arrest that eventually results in infertility^21^. In the context of spermatogenesis, peroxisomal *β*-oxidation is particularly required for the synthesis of docosahexaenoic acid (C22:6n-3; DHA), which is highly abundant in membrane phospholipids of round spermatids^22^ and mature spermatozoa^23^. DHA synthesis from linolenic acid is regulated by ELOVL2 and ELOVL5^24^. As a general by-product of both mitochondrial and peroxisomal *β*-oxidation, ROS are generated. Enhanced ROS levels can negatively influence the sperm concentration, motility and morphology leading to leukocytospermia, varicocele and idiopathic infertility^25^. Interestingly, mutations in *PEX13* have been shown to be linked to classical Zellweger syndrome, including intra-uterine growth retardation, hypotonia, abnormal peroxisomal metabolism and neonatal lethality^26^. Based on the *Cre*-*loxP* system, an *Amh-Cre* mediated *Pex13* knockout (KO) was formerly generated by our group to characterize peroxisomes exclusively in Sertoli cells. The *Pex13* KO induced a “Sertoli-cell-only” syndrome with a strong increase of neutral lipids, including triglycerides and cholesteryl esters^27^.

In the present study, we hypothesize that peroxisomal dysfunction in germ cells interferes with normal spermatogenesis, as peroxisomes provide essential metabolites to maintain cellular function. A conditional KO of one of the constituents of the translocation machinery, in our case PEX13, was specifically induced in pre-meiotic germ cells, using a transgenic Stra8*-Cre* promoter.

Our results show that truncated PEX13 abolished peroxisomal biogenesis leading to an impaired import of peroxisomal matrix proteins. Germ cell differentiation was interrupted at the round spermatid stage, resulting in the formation of MNCs and thus infertility of male mice. Peroxisomal genes involved in the metabolite transport, *β*-oxidation, ether lipid synthesis as well as retinoid and ROS metabolism were differently regulated in the *Pex13* KO. We also found alterations in the structural components of the BTB. With the present study, we provide initial data demonstrating that peroxisomes are necessary for spermiogenesis and indispensable for the maintenance of the tight junction barrier.

## Materials and Methods

### Generation of gc*Pex13*^∆ex2/∆ex2^/Stra8-Cre^+/−^ mice

*Pex13loxP* (*loxP*: locus of crossing over of the P1 phage) transgenic mice in C57Bl/6 J background were provided by Eveline Baumgart-Vogt. The germ cell-specific deletion of exon 2 of flanked *Pex13* was achieved by crossing homozygous male (or female) *Pex13*^*lox*P*/loxP*^ mice to corresponding female (or male) animals expressing *Cre* recombinase. *Cre* recombinase expression was directed by a STRA8 (stimulated by retinoic acid gene 8) genomic promoter fragment. Stra8*-Cre* transgenic animals in FVB/N background were obtained from Jackson laboratory (Bar Harbor, Maine, USA*)*. To generate a congenic mouse strain, mice were initially backcrossed into C57Bl/6 strain (Charles River Laboratories, Sulzfeld, Germany), based upon marker assisted selection protocol (MASP). Single nucleotide polymorphism (SNP) genotyping was carried out by LGC genomics GmbH (Herts, UK). After the fourth backcross into C57Bl/6 mice, the offspring were less than 1% original background strain and ≥ 99% C57Bl/6.

Heterozygous male (gc*Pex13*^*WT/*∆*ex2*^*/*Stra8*-Cre*^+/−^) from the F1 generation were crossed to homozygous (gc*Pex13*^*lox*P/loxP^) female mice to generate F2 offspring with the following genotypes: *Pex13*^*WT/loxP*^*/*Stra8*-Cre*^+/−^ (control; gc*Pex13*WT), *Pex13*^*WT/*∆*ex2*^*/*Stra8*-Cre*^+/−^ (heterozygous; gc*Pex13*HTZ) and gc*Pex13*^*∆ex2/∆ex2*^*/*Stra8*-Cre*^+/−^ (knockout; gc*Pex13*KO). Mouse tail biopsies were collected for DNA extraction, using the REDExtract-N-Amp Tissue PCR Kit (XNATS, Sigma-Aldrich, Missouri, USA) according to manufacturer´s instructions. The correct genotype was confirmed by appropriate primer pairs detecting the Stra8 driven *Cre* recombinase and flanked *Pex13* gene. All primers used for genotyping are listed in a supplemental Table T1.

For visualization of the peroxisomal compartment, GFP-PTS1 transgenic mice were crossed into heterozygous floxed *Pex13* mice carrying the Stra8*-Cre* transgene. In the GFP-PTS1 transgenic mice, a fusion protein of the green fluorescent protein (GFP) and PTS1 is frequently used for visualization of peroxisomes in living cells^28^.

The mouse line was generated in the laboratory of Professor Zimmer (Department of Neurobiology; University of Bonn, Germany) by injecting a GFP-PTS1 cDNA fragment under the control of the murine Rosa26 promoter into the pronucleus of CD1 mouse zygotes^29^.

All animals went through the embryo transfer at the transgenic animal facilities at the UKE Hamburg. Mice were housed under standard conditions with free access to standard laboratory food and water and a 12 hrs dark-/light-cycle. The use of mice was in accordance with the *Guide for the Care and Use of Laboratory Animals* from the Institute for Laboratory Animal Research. The experiment was supervised by the institutional animal welfare officer and approved by the local licensing authority (Behörde für Soziales, Familie, Gesundheit, Verbraucherschutz; Amt für Gesundheit und Verbraucherschutz; Billstr. 80, D-20539 Hamburg, Germany). All methods were performed in accordance with the relevant guidelines and regulations by the local authorities.

### Processing of testes for cryo and paraffin embedding and sectioning

Mice were anaesthetized by intraperitoneal injection using a cocktail of 100 mg/kg ketamine and 10 mg/kg xylazine and euthanized by cervical dislocation. Testicles were aseptically removed from the scrotum. The *Tunica albuginea* was carefully dissected and testicles were perfused with 4% paraformaldehyde (PFA; pH 7.4). For paraffin embedding, testes were immersed in 4% PFA at 4 °C overnight. Testes were transferred into phosphate buffer until embedded into paraffin (Paraplast, Sigma-Aldrich, Missouri, USA). Paraffin blocks of testes were cut into sections of 2–4 µm thickness, using a Leica rotation microtome RM 2135. For cryo-sections, biopsies were immersed in 30% (w/v) sucrose solution at 4 °C overnight. The tissue was frozen in isopentane at −30 °C and stored at −80 °C. Cryo-sections of about 8–10 µm were cut using a cryostat (Leica Biosystems, Wetzlar, Germany).

### Hematoxylin and eosin staining of testis sections

Paraffin sections (2 µm) of adult mouse testes from all genotypes were stained with hematoxylin and eosin (HE) in an automated system. The sections were examined with a ZEISS microscope (AxioVision, Oberkochen, Germany).

### Indirect immunofluorescence

Deparaffinized and rehydrated testis sections were subjected to digestion with 0.1% trypsin (Biochrom, Berlin Germany) for 7 min at 37 °C, followed by microwave treatment for 3 × 5 min at 900 W in 10 mM citrate buffer at pH 6.0 (modified according to Grabenbauer *et al*.)^30^. Nonspecific binding sites were blocked with 4% BSA in PBS for 2 hrs at room temperature, and sections were incubated with primary antibodies in 1% BSA in TBS-T for 1 hr. Following primary antibodies were used: anti-ABCD3 (rabbit, polyclonal), anti-BAX (D3R2M; rabbit, monoclonal; #14796; Cell Signaling Technology, Cambridge, UK), anti-Catalase (rabbit, polyclonal), anti-OSP/Claudin-11 (rabbit, polyclonal; #36–4500; Invitrogen, Carlsbad, USA), anti-Cleaved Caspase-3 (rabbit, polyclonal; #9661; Cell Signaling Technology, Cambridge, UK), anti-PEX13 (rabbit, polyclonal) and anti-PEX14 (rabbit, polyclonal), anti-Thiolase (rabbit, polyclonal). The secondary antibodies for visualization of immune complexes were AlexaFlour488 (donkey anti-rabbit; Invitrogen, Carlsbad, USA), Cy3 (goat anti-mouse; Jackson Immuno Research, West Grove, USA) and Cy3 (donkey anti-rabbit; Jackson Immuno Research, West Grove, USA). Negative controls were processed in parallel. For counterstaining of nuclei, the secondary antibody was supplemented with 1 µg/ml 4′,6-Diamidino-2-phenylindole (DAPI; Thermo Fisher Scientific, Massachusetts, USA). Specimens were mounted with Mowiol 4–88 (Roth, Karlsruhe, Germany). Specimens were analyzed using a Confocal Laser Microscope (Nikon) with standard filters for detection of Cy3, Alexa488 and DAPI. Digital images were obtained with a Nikon A1plus camera using the Nikon NIS Elements Advanced Research software.

### Electron microscopy of testis sections

Mice testes were fixed by perfusion with 4% depolymerized PFA (w/v, pH 7.4). Dissected testes fragments were post-fixed with 5.5% glutaraldehyde (Merck, Darmstadt, Germany) in 0.05 M phosphate buffer (v/v) overnight. Testes fragments were further treated with 1% aqueous osmium tetroxide (Roth, Karlsruhe, Germany) for 90 min and subjected to dehydration in a series of graded alcohol (35% to absolute) before they were embedded in a mixture of 1,2,3-propanetriol glycidyl ether (Epon) and propylene oxide (Serva, Heidelberg, Germany). 1 μm semithin sections were cut to determine the region of interest. For electron microscopy, 80 nm ultrathin sections were cut. The sections were examined on an electron microscope (Philips, Amsterdam, Netherlands).

### Oil Red O staining

Cryo-sections (6 μm) of testes were stained with Oil red O (ORO; Sigma-Aldrich, Missouri, USA) for the detection of lipid droplets. ORO staining was performed according to the protocol of Lillie *et al*. (1943) using a 0.5% ORO stock solution in isopropanol^31^. Nuclei were counterstained with Mayer’s hematoxylin (5 dips for 5 s) and rinsed thereafter with distilled water. The stained sections were mounted in Mowiol 4–88 (Roth, Karlsruhe, Germany).

### Lipid analysis by gas chromatography

Total testicular triglyceride and phospholipid levels were quantified by gas chromatography. Tissue lipid extracts were prepared according to Folch *et al*. (1957) and total tissue fatty acid profiling was performed as already described^32^, using 20 µl of solvent per mg of tissue. Lipid classes were separated on silica gel 60 plates: 100 µl of extract was spotted onto the plate and developed with an eluent containing hexane, diethylether and acetic acid (80:20:1.5). Visualization of lipid bands was performed with primuline (5 mg in 100 ml acetone:water 80:20). Fatty acid methyl esters were prepared from 25 µl Folch extract (total FA) or of the scratched bands of PL and TG fractions without further extraction, based on the method of Lepage and Roy^33^ by adding 1 ml methanol/toluene (4:1), 100 μl heptadecanoic acid (200 μg/ml in methanol/toluene, 4:1), 100 μl acetyl chloride and heating in a capped tube for 1 hr at 100 °C. After cooling to room temperature, 3 ml of 6% sodium carbonate were added. The mixture was centrifuged (1800 × g, 5 min.). 5 μl of the upper layer was diluted 1:5 with toluene and transferred to auto sampler vials. Gas chromatography analyses were performed using an HP 5890 gas chromatograph (Hewlett Packard) equipped with flame ionization detector (Stationary phase: DB-225 30 m × 0.25 mm id., film thickness 0.25 μm; Agilent, Böblingen, Germany). Peak identification and quantification were performed by comparing retention times, respectively, peak areas to standard chromatograms. All calculations are based on fatty acid methyl esters values. Concentration of individual fatty acids was calculated as % of total fatty acids.

### Evans Blue

The azo dye Evans Blue (Sigma-Aldrich, Missouri, USA) was applied by an intravenous injection at a dose of 100 µl (50 mg/ml PBS). Adult mice of all genotypes were used. The azo dye was circulating in the blood system for 1 hr until mice were anaesthetized and intracardially perfused with 1% BSA in NaCl to remove free dye. Testes were surgically removed and fixed overnight in an additional step of 3.7% buffered formalin. For cryo-preservation, the samples were first transferred to 15% sucrose (30 min), followed by 30% sucrose (30 min) and stored at −80 °C. Prior to use, tissue samples were directly embedded into a cryo-preservative solution (Optimal Cutting Temperature, OCT, Tissue-tek^®^). Cryo-sections of 6 µm thickness were obtained on a LEICA microtome (CM3050; Wetzlar, Germany). The level of incorporated Evans Blue was assessed at 620 nm using a confocal laser microscope (Nikon Eclipse Ti NIS-Elements).

### Total RNA isolation from testes biopsies

Total RNA from testes was isolated by phenol-chloroform extraction. Testes biopsies were taken from all genotypes. The *Tunica albuginea* was removed. Decapsulated testicles were immediately transferred to liquid nitrogen. Cooled mortar and pistil were used to mince the tissue. 1 ml TRIzol (Qiagen, Hilden, Germany) was added to the pulverized tissue and kept at room temperature. Homogenate was transferred to an Eppendorf tube and chloroform was added at one fifth of the total volume. After centrifugation (14000 x g, 20 min at 4 °C), the upper liquid phase was carefully removed and transferred to another Eppendorf tube. RNA was precipitated with isopropyl alcohol, followed by cooling at −20 °C for 30 min and centrifugation (14000 x g, 20 min at 4 °C). The pellet was washed two times with 70% ethanol and solubilized in 100 µl RNase-free water. RNA concentrations were quantified using a nanodrop ND-1000 spectrophotometer (Thermo Fisher Scientific, Massachusetts, USA).

### QRT-PCR and primers used

For cDNA synthesis, purified RNA of whole testes was reverse transcribed with the RT^2^ First Strand Kit (Qiagen, Hilden, Germany). Real-time PCR amplification of genes of either cell-cell junctions or fatty acid synthesis was performed on 96-well plates in a LightCycler^®^ 480 Real-Time PCR System (Roche, Basel, Switzerland). The mRNA level of peroxisome-related genes was evaluated with a customized RealTime ready Panel in a 384 multi-well plate format (Roche, Basel, Switzerland). QRT-PCR was performed according to manufacturer´s instructions using the SYBR Green I Master kit. 5 ng of cDNA were applied per reaction. The samples were run in triplicate following the MIQE guidelines^34^. A no-template control with RNase-free water instead of cDNA was included. Expression levels were calculated as relative values using the mean of both reference genes *Gapdh* and *β-actin*. The difference of the C_T_-values (ΔC_T_) from the target gene and the mean of the C_T_-values from both reference genes were determined to quantify the target gene expression. Expression levels were further related (ΔΔC_T_) to control samples using the difference of the ΔC_T_ -value from the sample (ΔC_T_ sample) as well as the ΔC_T_ -value from the control (ΔC_T_ control) and the relative values were calculated as the 2^^−ΔΔCT^ according to Livak and Schmittgen (2001)^35^. Oligonucleotides are listed in a supplemental Table T1.

### Statistical analysis

GraphPad Prism v.5 software was used for statistical analyses. Statistically significant differences between groups were determined using Student’s t-test. A p value < 0.05 was considered statistically significant.

## Results

### Impaired spermatogenesis in gc*Pex13*^∆ex2/∆ex2^/Stra8-Cre^+/−^ mice led to infertility of male mice

No abnormalities of external genitalia or differences in the epididymis, deferent ducts, seminal vesicles or prostate glands were detected in male gc*Pex13*^*∆ex2/∆ex2*^*/*Stra8*-Cre*^+/−^ mice. However, when male gc*Pex13*^*∆ex2/∆ex2*^*/*Stra8*-Cre*^+/−^ mice were tested for fertility through mating with fertile wildtype (WT) females, females produced no offspring, indicating that the male gc*Pex13*^*∆ex2/∆ex2*^*/*Stra8*-Cre*^+/−^ mice were sterile.

**Figure 1:**
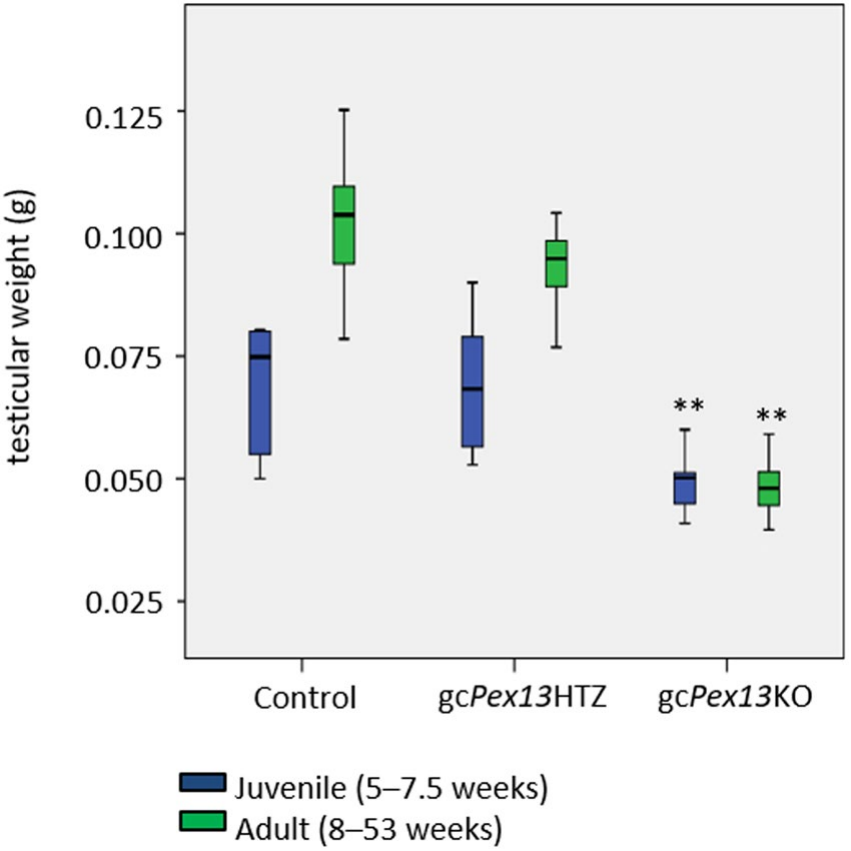
Analyses of testicular weight of all genotypes of juvenile (blue bars) and adult (green bars) mice. gc*Pex13*^*∆ex2/∆ex2*^*/*Stra8*-Cre*^+/−^ (gc*Pex13*KO) testes of juvenile and adult mice showed a significant reduction in their weight compared to *Pex13*^*WT/*∆*ex2*^*/*Stra8*-Cre*^+/−^ (gc*Pex13*HTZ) and control testes. For data collection, a minimum of 30 individual testicles from adult mice and a minimum of 4 testicles from juvenile animals were analyzed. *p ≤ 0.05; **p 0.001 < p ≤ 0.01; *** p ≤ 0.001.

The gross morphology of gc*Pex13*^*∆ex2/∆ex2*^*/*Stra8*-Cre*^+/−^, heterozygous and control testes was subsequently analyzed comparing juvenile (5–7.5 weeks) with adult mice (8–53 weeks). For data collection, a minimum of 30 individual testicles from adult mice and a minimum of 4 testicles from juvenile animals were analyzed. The total testis weight with 0.046 ± 0.008 g of adult gc*Pex13*^*∆ex2/∆ex2*^*/*Stra8*-Cre*^+/−^ mice was significantly reduced (p ≤ 0.01) compared to control with 0.101 ± 0.012 g and heterozygous mice with 0.093 ± 0.010 g. Testes weight of juvenile control testes was 0.069 ± 0.014 g, whereas the average weight of gc*Pex13*^*∆ex2/∆ex2*^*/*Stra8*-Cre*^+/−^ testes from juvenile mice was slightly decreased with 0.049 ± 0.005 g (Fig. 1). The lengths of gc*Pex13*^*∆ex2/∆ex2*^*/*Stra8*-Cre*^+/−^ testes from adult mice were significantly reduced to 0.442 ± 0.311 cm compared to the testicle length of 0.780 ± 0.076 cm of control and heterozygous mice (0.764 ± 0.075 cm).

**Figure 2:**
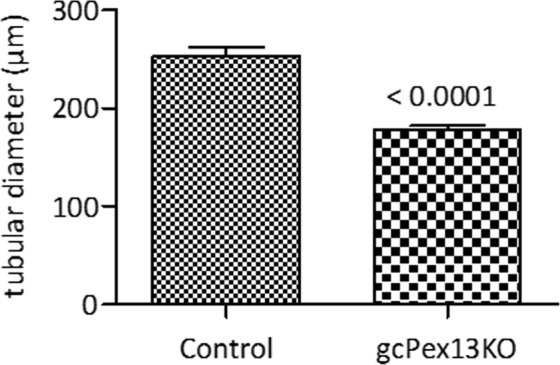
Comparison of the average size of the seminiferous tubules of control and gc*Pex13*^*∆ex2/∆ex2*^*/*Stra8*-Cre*^+/−^ (gc*Pex13*KO) testes. The size of the seminiferous tubules of the gc*Pex13*^*∆ex2/∆ex2*^*/*Stra8*-Cre*^+/−^ was significantly reduced (p < 0.0001) compared to control testes. Statistical significance was determined using t-test (non-parametric Mann-Whitney *U* test). For the evaluation, 15 seminiferous tubules of 3 animals per group, respectively, were considered.

**Figure 3:**
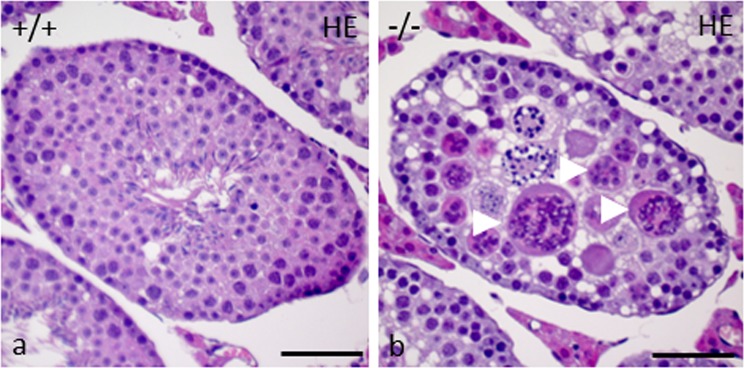
Representative HE staining of paraffin embedded cross-sections of seminiferous tubules at stage VIII of control (+/+) and gc*Pex13*^*∆ex2/∆ex2*^*/*Stra8*-Cre*^+/−^ (−/−) testes from adult mice (P360). (**a)** The germinal epithelium of control testes showed regular spermatogenesis with meiotically dividing spermatocytes, spermatids and spermatozoa. (**b)** In the gc*Pex13*^*∆ex2/∆ex2*^*/*Stra8*-Cre*^+/−^ testis, spermatid nuclei were arranged as MNCs (indicated by *arrowheads*). Bars = 0 µm.

For the examination of phenotypic characteristics, seminiferous tubules of adult mice aged from 8 to 52 weeks were analyzed. Compared to control testes (253.1 ± 33.0 µm), the diameter of the seminiferous tubules of gc*Pex13*^*∆ex2/∆ex2*^*/*Stra8*-Cre*^+/−^ mice was significantly smaller (177 ± 18.82 µm; p < 0.0001; Fig. 2) with increased number of interstitial cells (hyperplastic). In total, 15 seminiferous tubules of 3 animals per group were included into the evaluation. As representatives for the histological analyses, stage VII seminiferous tubules of adult (P360) mice were considered. Whereas normal littermates displayed spermatogenic cells at all stages, including spermatozoa (Fig. 3a), a severe defect in germ cell differentiation in gc*Pex13*^*∆ex2/∆ex2*^*/*Stra8*-Cre*^+/−^ testes was observed in HE stained paraffin sections. Instead of single round spermatids, spermatid stage nuclei were arranged as MNCs (Fig. 3b), similar to those described by MacGregor^36^. Their average size was 39.83 ± 8.78 µm. Germ cell-specific gene expressions were quantified in isolated germ cells to confirm the spermatogenic arrest at the round spermatid stage in gc*Pex13*^*∆ex2/∆ex2*^*/*Stra8*-Cre*^+/−^ testes. The spermatocyte-specific marker *Sycp3* and late spermatid markers, including *Prm1* and *Acrv1*, were significantly down-regulated in the gc*Pex13*^*∆ex2/∆ex2*^*/*Stra8*-Cre*^+/−^ testes compared to control mice (Table 1).

**Table 1:** Expression profiles of peroxisomes-related genes in testicular cells of whole testes biopsies from control and gc*Pex13*^*∆ex2/∆ex2*^*/*Stra8*-Cre*^+/−^ (gc*Pex13*KO) mice.

Function	Gene	Control	gc*Pex13*KO	*p-*Value
**Cell-type specific marker**				
Spermatocytes	*Sycp3*	1.05 ± 0.20	0.07 ± 0.04	0.0406
Spermatids	*Prm1*	1.04 ± 0.21	0.01 ± 0.00	0.0386
	*Acrv1*	1.00 ± 0.03	0.01 ± 0.01	0.0008
**Peroxisomal genes**				
Translocation machinery	*Pex13*	1.03 ± 0.20	20.88 ± 15.32	0.3245
	*Pex14*	1.05 ± 0.20	0.26 ± 0.12	0.0425
Peroxisome membrane assembly	*Pex19*	1.14 ± 0.37	0.35 ± 0.18	0.1935
ABC transporters	*Abcd1*	1.06 ± 0.23	0.09 ± 0.05	0.0559
Fatty acid *β*-oxidation	*Acox1*	1.03 ± 0.18	0.41 ± 0.19	0.0976
	*Hsd17b4*	1.25 ± 0.49	0.12 ± 0.07	0.1510
ROS metabolism	*Cat*	1.25 ± 0.59	0.03 ± 0.02	0.1739
Superoxide dismutase	*Sod1*	1.02 ± 0.14	0.18 ± 0.09	0.0149

Relative expression values were calculated by the ΔΔ*C*_*T*_-method. The qRT-PCR data of 3 biological replicates per group (including 3 technical replicates each) were calculated and represented as a mean ± SD. Statistical significance was determined using t-test (Welch´s correction). *p ≤ 0.05; **p0.001 < p ≤ 0.01; ***p ≤ 0.001.

**Figure 4:**
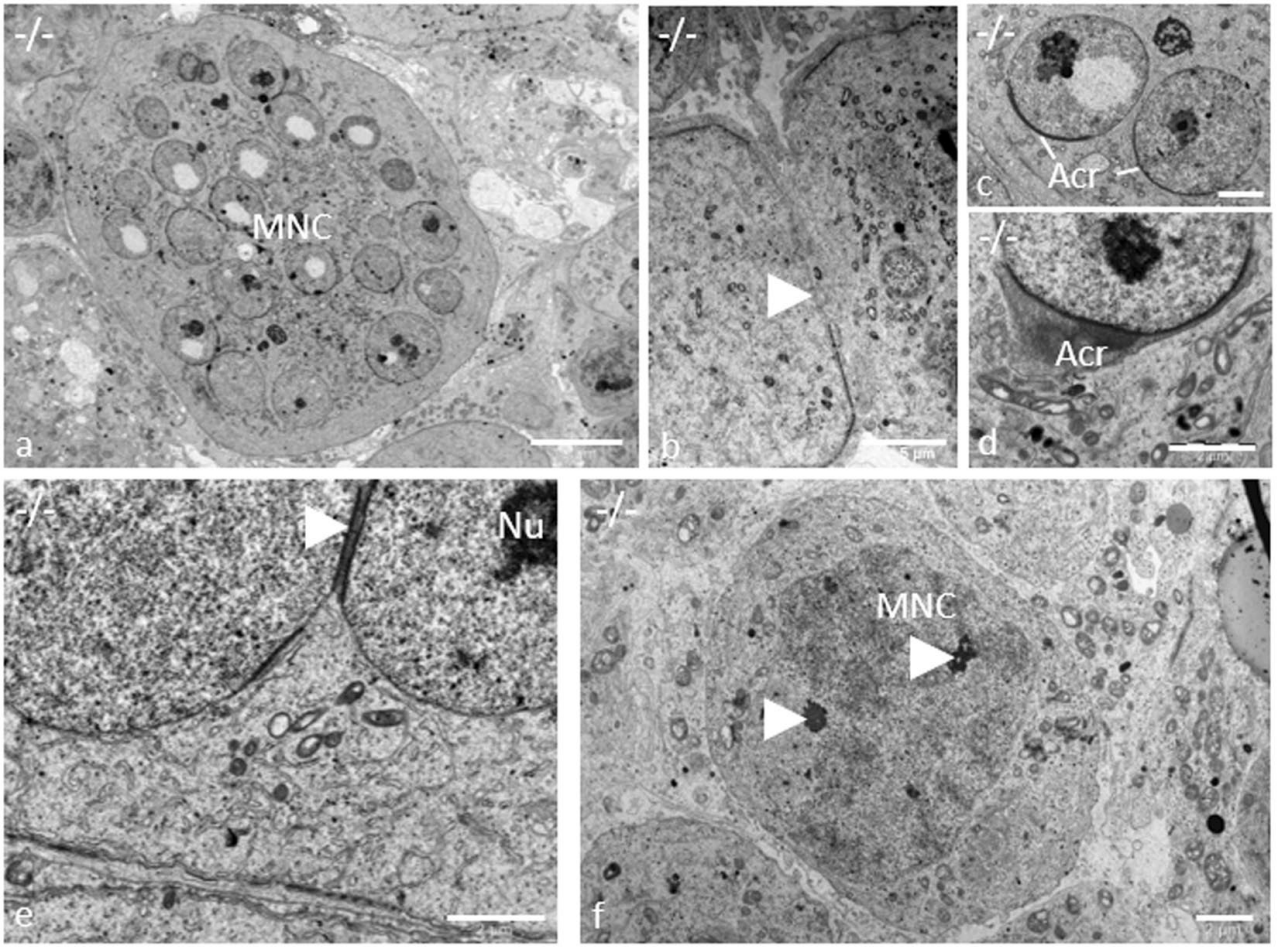
MNCs in gc*Pex13*^*∆ex2/∆ex2*^*/*Stra8*-Cre*^+/−^ (−/−) testes of adult mice (11 weeks). (**a)** Round spermatids were arranged as MNCs with up to 32 nuclei. (**b)** MNCs were in close contact with neighboring MNCs, linked over intercellular bridges (indicated by *arrowheads*). (**c**, **d)** Round spermatids initiated acrosome (Acr) formation. (**e)** Some acrosomes were shared by two or even more spermatid nuclei (indicated by *arrowheads*). (**f)** At stage IX, spermatid nuclei were condensed (indicated by *arrowheads*), as indication for apoptosis. Acr: Acrosome; MNC: Multinucleated giant cells; Nu: Nucleus. (**a)** Bar = 10 µm. (**b)** Bar = 5 µm. (**c–f)** Bars = 2 µm.

**Figure 5:**
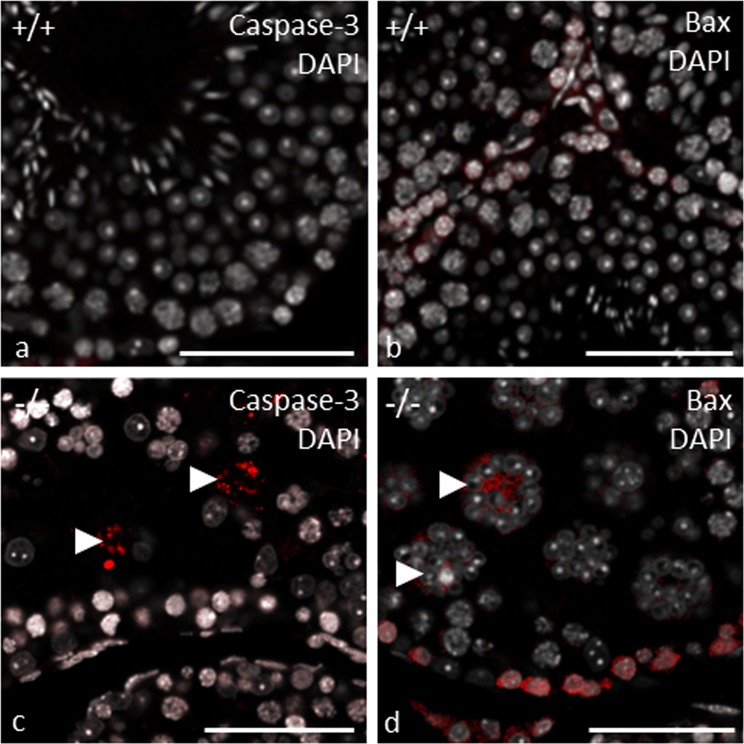
Immunofluorescent detection of apoptotic markers in germ cells of control (+/+) and gc*Pex13*^*∆ex2/∆ex2*^*/*Stra8*-Cre*^+/−^ (−/−) testes of adult mice (8 weeks). (**a**,**b)** In control testes, some spermatogonia and spermatocytes were positive for **(a)** Caspase-3 and **(b)** BAX. (**c**,**d)** In gc*Pex13*^*∆ex2/∆ex2*^*/*Stra8*-Cre*^+/−^ mice, spermatogonia and spermatocytes were labelled with **(c)** anti-Cleaved Caspase-3 and **(d)** anti-BAX antibodies. **c)** MNCs were also positive for Caspase-3 and **(d)** BAX (indicated by *arrowheads*). Nuclei were counterstained with DAPI (grey). Bars = 50 µm.

Pathological alterations in the germinal epithelium of gc*Pex13*^*∆ex2/∆ex2*^*/*Stra8*-Cre*^+/−^ testes from adult mice (11 weeks) were subsequently analyzed at ultrastructural level. Strikingly, all MNCs contained vast amounts of cytoplasm (Fig. 4a). Intercellular bridges did not seem to be affected, as MNCs were still in close contact to neighboring MNCs (Fig. 4b). All spermatids started to form an acrosomic system (Fig. 4c,d). In some cases, one or more spermatid nuclei even shared an acrosome (Fig. 4e). With initiation of the transition into elongated spermatids at stage IX, nuclei of MNCs became more condensed, as an early sign of beginning apoptosis (Fig. 4f). The dynamic expression of programmed cell death-related proteins was confirmed by anti-BAX and anti-Cleaved Caspase-3 antibodies. In control tubules, cell death was mainly identified in meiotically dividing germ cells, as already described^37^ (Fig. 5a,b). In gc*Pex13*^*∆ex2/∆ex2*^*/*Stra8*-Cre*^+/−^ testes, a similar pattern of apoptotic cells in the germinal epithelium was observed. Regarding the spermatogenic state, some MNCs, as well as dividing spermatogonia and spermatocytes were positive for Cleaved Caspase-3 (Fig. 5c) and Bax in the gc*Pex13*^*∆ex2/∆ex2*^*/*Stra8*-Cre*^+/−^ testes (Fig. 5d).

### Peroxisomal proteins showed a different distribution in the gc*Pex13*^∆ex2/∆ex2^/Stra8-Cre^+/−^ testes

**Figure 6:**
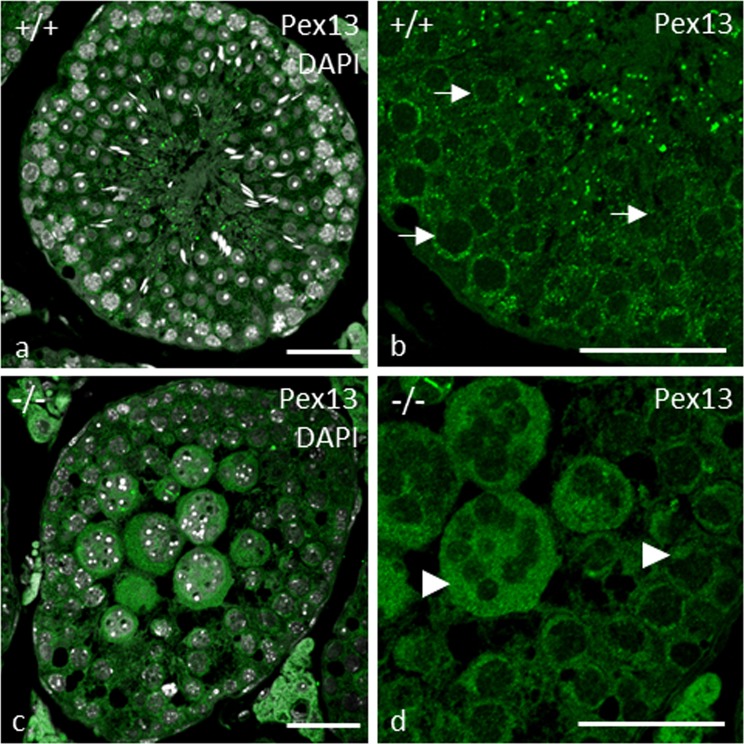
Immunofluorescent visualization of the peroxisomal membrane protein PEX13 in control (+/+) and gc*Pex13*^*∆ex2/∆ex2*^*/*Stra8*-Cre*^+/−^ (−/−) testes of adult mice (8 weeks). (**a**,**b)** Localization of PEX13 (green) in control testes showed a for peroxisomes typical pattern in Sertoli cells and all germ cells (indicated by *arrows* (**b**) in the higher magnification), except for spermatozoa. (**c**,**d)** In *Pex13*-deficient cells, peroxisomal remnants were found (indicated by *arrowheads* (**d**) in the higher magnification). All preparations were counterstained with DAPI (grey) for labelling of nuclei. Bars = 50 µm.

To confirm the *Pex13* KO, we performed indirect immunofluorescent analyses in testes from adult mice (8 weeks). In the seminiferous tubules of control testes, immunoreactivity for anti-PEX13 was shown in testis-specific somatic cells (Sertoli cells and peritubular cells) and germ cells (spermatogonia, primary and secondary spermatocytes, round and elongated spermatids), with the exception for mature spermatozoa in these mice. Pex13 immunoreactivity was most intense in spermatocytes and spermatids but weaker in Sertoli-, peritubular myoid and Leydig cells (Fig. 6a,b). In germ cells with a *Pex13* KO, PEX13 was not localized as punctuate structures (Fig. 6c,d). Surprisingly, gene expression analysis showed a highly significant up-regulation of *Pex13* mRNA in gc*Pex13*^*∆ex2/∆ex2*^*/*Stra8*-Cre*^+/−^ mice (Table 1). Note, that the primers for the quantification of *Pex13* mRNA were designed to specifically anneal in exon 4, which was not affected by the *Cre*-mediated excision.

**Figure 7:**
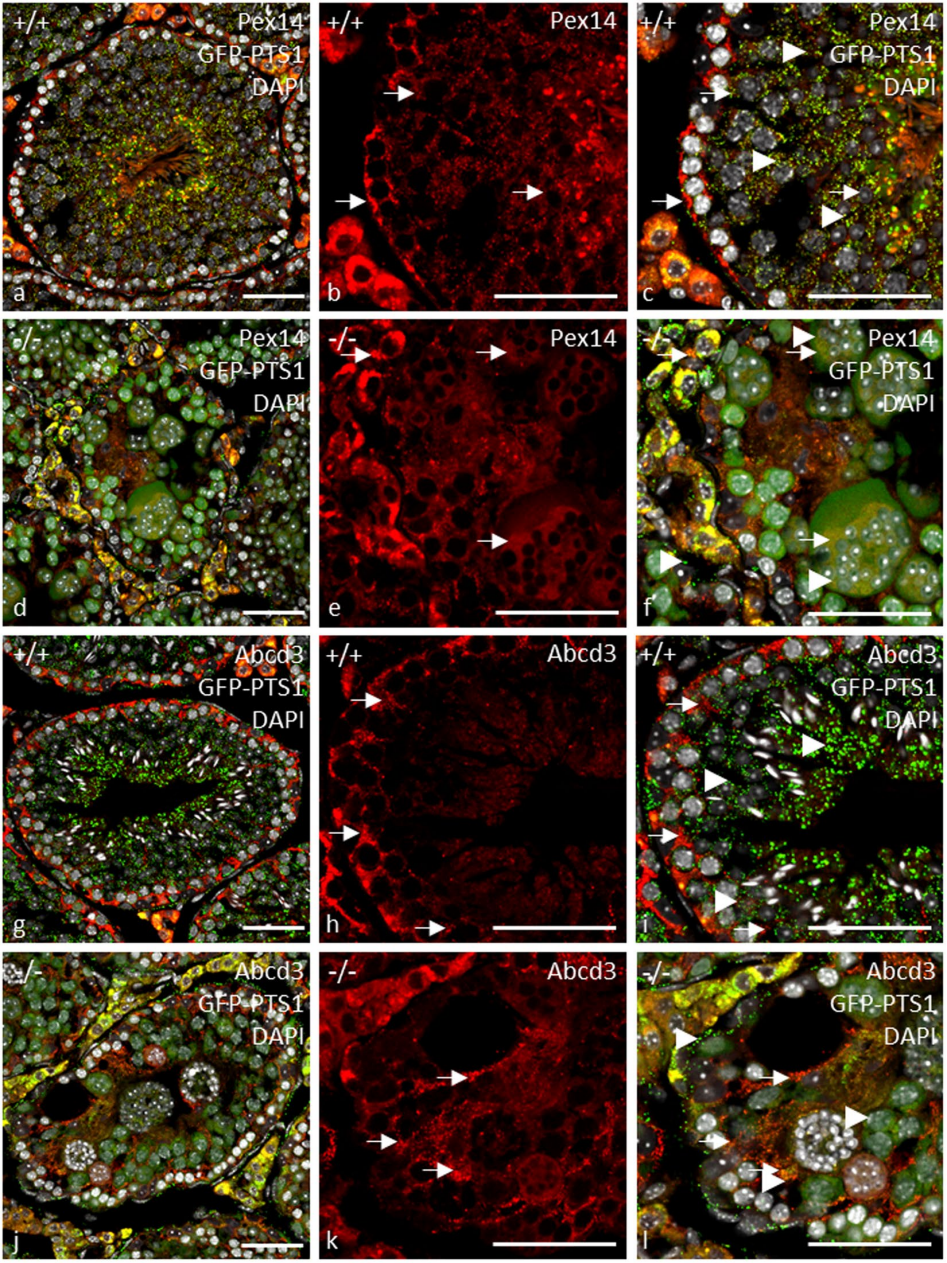
Immunofluorescence analyses of peroxisomal membrane proteins in control (+/+) and gc*Pex13*^*∆ex2/∆ex2*^*/*Stra8*-Cre*^+/−^ (−/−) testes of adult mice (8 weeks). Peroxisomal membrane proteins: (**a–f)** PEX14 (red) and (**g–l**) ABCD3 (red) immunoreactivity in cryo-sections of GFP-PTS1 (green) transgenic mouse testes. Note the almost complete co-localization of peroxisomal membrane proteins with the GFP-PTS1 fusion protein in control testes (**a**,**c**,**g**,**i**). In *Pex13*-deficient cells, GFP was only detected in peritubular myoid cells, in the basal epithelium and in Leydig cells. The distribution of all membrane proteins was clearly cytoplasmic in all germ cells, including MNCs (**d**,**f**,**j**,**l**). The peroxisomal membrane proteins are indicated by arrows. The GFP-PTS1 fusion protein is indicated by *arrowheads*. All preparations were counterstained with DAPI (grey) for labelling of nuclei. Bars = 50 µm.

The existence of peroxisomal remnants in *Pex13-*deficient cells was confirmed in GFP-PTS1 transgenic gc*Pex13*^*∆ex2/∆ex2*^*/*Stra8*-Cre*^+/−^ mice. Whereas the GFP-PTS1 fluorescence pattern within the germinal epithelium of control mice showed characteristic peroxisomal structures (Fig. 7a,c,g,i), we found a typical expression of the GFP-PTS1 transgene in peritubular myoid cells, in the basal epithelium and in Leydig cells but not in MNCs of gc*Pex13*^*∆ex2/∆ex2*^*/*Stra8*-Cre*^+/−^ mice (Fig. 7d,f,j,l). Although the presence of peroxisomes was already confirmed in Sertoli cells, GFP was not expressed in these cells (Fig. 7c,f,i,l). As PEX13 directly interacts with the core protein PEX14, the effects of the *Pex13* KO on the translocation machinery were further substantiated in GFP-PTS1 transgenic gc*Pex13*^*∆ex2/∆ex2*^*/*Stra8*-Cre*^+/−^ mice by fluorescence analysis, using anti-PEX14. In control testes, PEX14 co-localized with the GFP-PTS1 transgene and was detected in all germ cells, peritubular myoid and Leydig cells (Fig. 7a–c). However, in *Pex13*-deficient cells of gc*Pex13*^*∆ex2/∆ex2*^*/*Stra8*-Cre*^+/−^ testes, PEX14 was not found as punctuate structures (Fig. 7d–f). In concordance to that, *Pex14* mRNA was significantly lowered in the gc*Pex13*^*∆ex2/∆ex2*^*/*Stra8*-Cre*^+/−^ testes (Table 1). The expression of the peroxisomal biogenesis factor *Pex19* was likewise but not significantly decreased (Table 1). To test for a defect in the import of metabolites including long-chain unsaturated acyl-CoAs, 2-methyl branched-chain acyl-CoAs and long-chain dicarboxylic CoA esters, the localization of the peroxisomal ABC transporter ABCD3 (70 kDa peroxisomal membrane protein; PMP70) was analyzed at protein level. In control seminiferous tubules, ABCD3 showed immunoreactivity to peroxisomes in Leydig cells, Sertoli cells and in germ cells of the basal part of the germinal epithelium (Fig. 7g–i). In MNCs of gc*Pex13*^*∆ex2/∆ex2*^*/*Stra8*-Cre*^+/−^mice, ABCD3 was not localized in a typical punctuate pattern (Fig. 7j–l). In qRT-PCR analyses, the metabolite transporter gene *Abcd1*, whose mutated form is related to X-ALD, was strongly but not significantly down-regulated.

*Acox1*, that is involved in the dehydrogenation step during *β*-oxidation, together with the gene that expresses the bifunctional enzyme involved in the peroxisomal *β*-oxidation pathway for fatty acids, *Hsd17b4*, were likewise down-regulated in the gc*Pex13*^*∆ex2/∆ex2*^*/*Stra8*-Cre*^+/−^ testes compared to control (Table 1).

**Figure 8:**
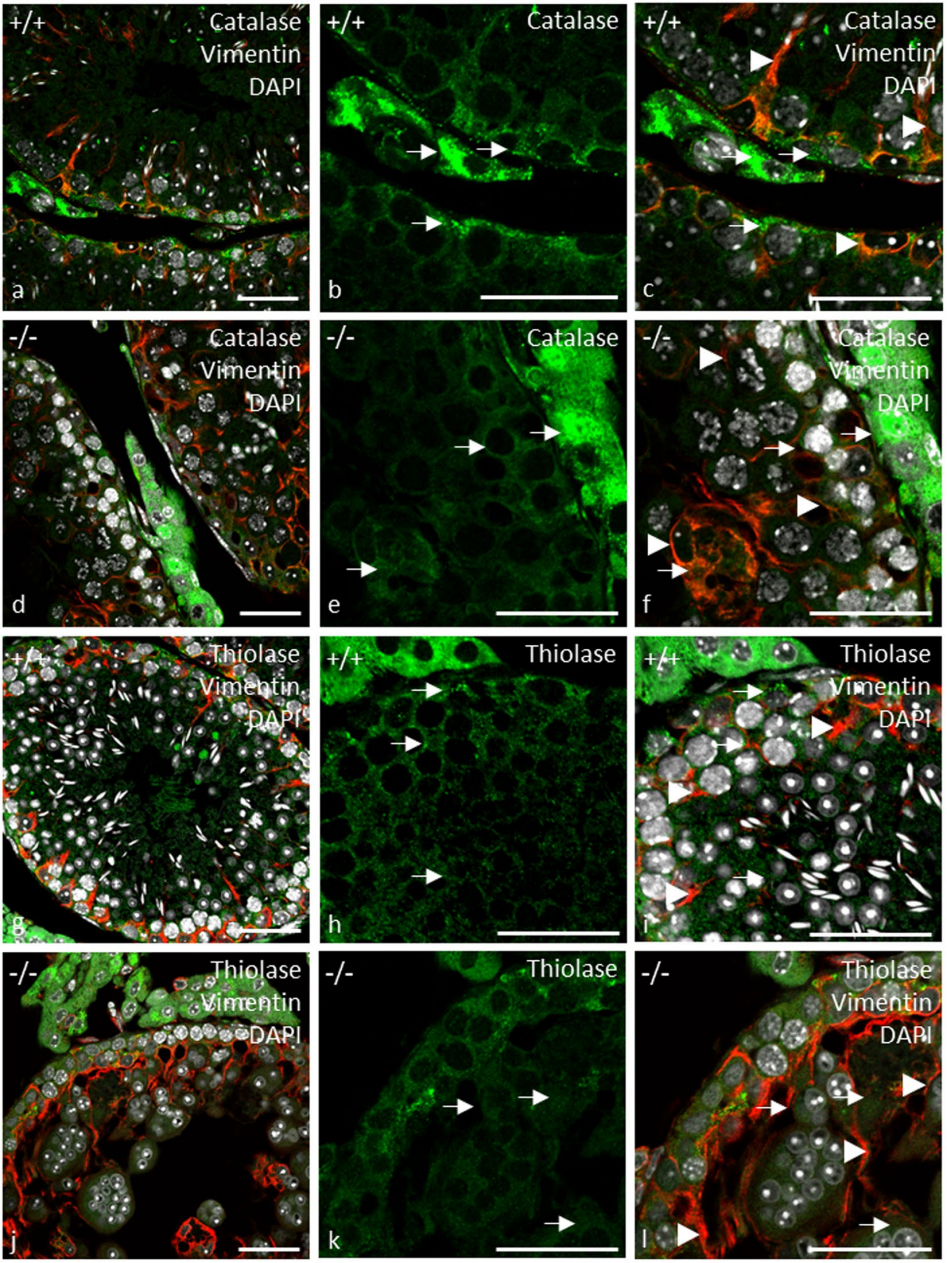
Immunofluorescence analyses of peroxisomal matrix proteins in control (+/+) and gc*Pex13*^*∆ex2/∆ex2*^*/*Stra8*-Cre*^+/−^ (−/−) testes of adult mice (8 weeks). (**a–f)** Double-immunofluorescent labelling for catalase (green) and (**g–l**) thiolase (green) with Sertoli cell marker vimentin (red). (**a–c**,**g–i)** In control testes, the matrix proteins were shown as punctuate structures. (**d–f**,**j–l)** In *Pex13*-deficient cells, peroxisomal matrix proteins did not show a typical peroxisomal pattern. (**b**,**e**,**h**,**k)** The matrix proteins are shown in a higher magnification. (**f**,**l)** In the gc*Pex13*KO testes, vimentin immunostaining showed an irregular pattern of intermediate filaments of Sertoli cells. The matrix proteins are indicated by *arrows*. The Sertoli cell marker vimentin is indicated by *arrowheads*. All preparations were counterstained with DAPI (grey) for labelling of nuclei. Bars = 50 µm.

The localization of thiolase, which is an acetyl-coenzyme A acetyltransferase of the peroxisomal *β*-oxidation, was further assessed by an immunofluorescent staining to assay effects on peroxisomal matrix import. Thiolase showed strong immunoreactivity in interstitial Leydig cells and peritubular myoid cells of control (Fig. 8g–i) and gc*Pex13*^*∆ex2/∆ex2*^*/*Stra8*-Cre*^+/−^ testes (Fig. 8j–l). However, in *Pex13-*deficient germ cells of gc*Pex13*^*∆ex2/∆ex2*^*/*Stra8*-Cre*^+/−^ testes, thiolase showed an atypical pattern (Fig. 8j–l), thereby indicating a defect in the import of peroxisomal proteins. Impaired translocation of matrix proteins was further confirmed by a double-immunofluorescent staining with catalase and the Sertoli cell intermediate filament marker vimentin. Signal intensity of catalase was most intense in Leydig cells and peritubular myoid cells of control (Fig. 8a–c) and gc*Pex13*^*∆ex2/∆ex2*^*/*Stra8*-Cre*^+/−^ (Fig. 8d–f) testes. In *Pex13*-deficient germ cells of gc*Pex13*^*∆ex2/∆ex2*^*/*Stra8*-Cre*^+/−^ testes, the catalase protein was mistargeted to the cytoplasm and not localized in peroxisomes (Fig. 8d–f). On mRNA level, catalase together with the gene, encoding the antioxidant enzyme superoxide dismutase (*Sod1*), were strongly down-regulated in the gc*Pex13*^*∆ex2/∆ex2*^*/*Stra8*-Cre*^+/−^ testes compared to control mice (Table 1).

### Tight junction proteins were affected by the germ cell-specific *Pex13* KO

**Figure 9:**
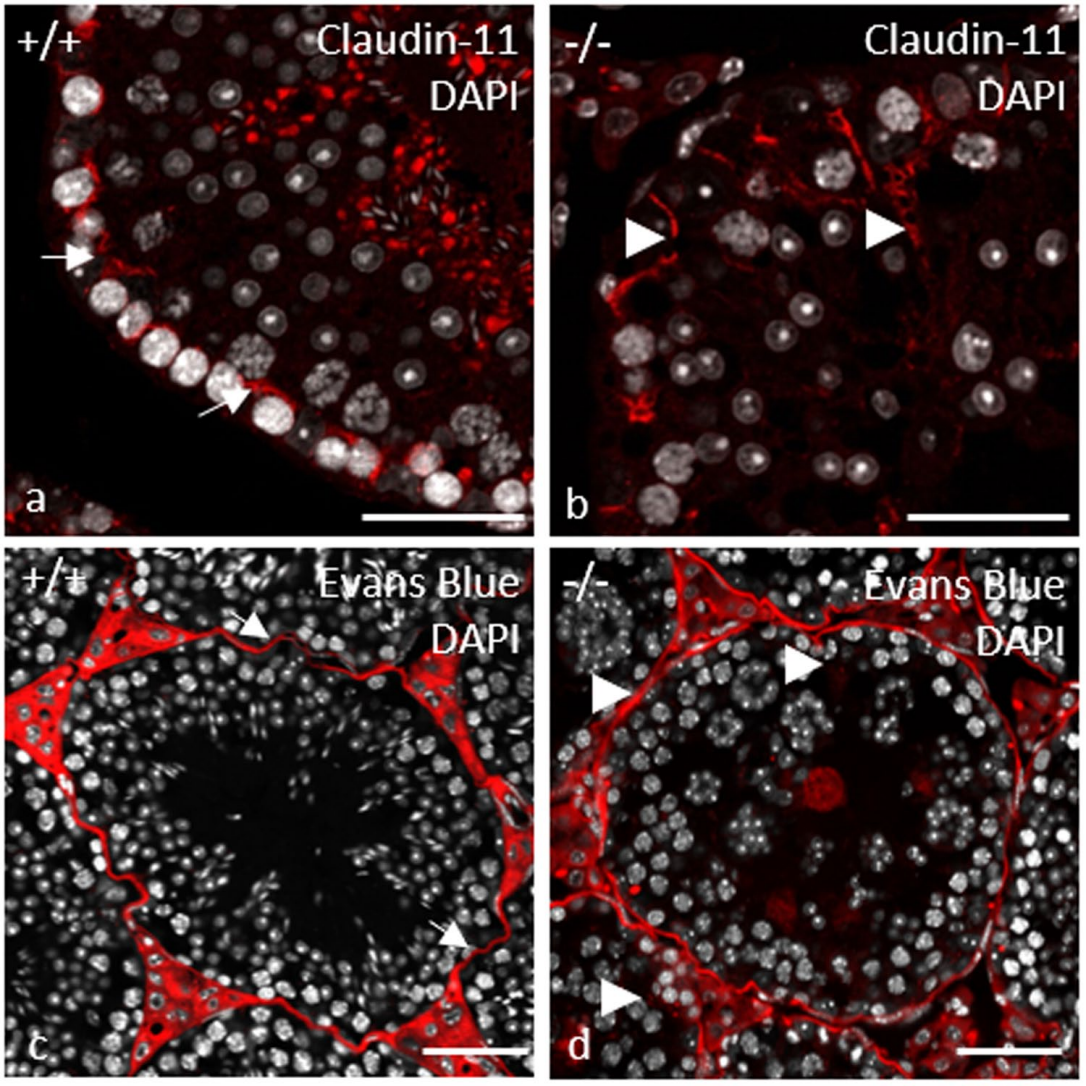
Immunofluorescence staining against claudin-11 reveals a structural disturbance of the BTB (**a**,**b**) in gc*Pex13*^*∆ex2/∆ex2*^*/*Stra8*-Cre*^+/−^ testes that was functionally assayed by Evans Blue injection (**c**,**d**). (**a)** Claudin-11 (red) was concentrated in the suprabasal layer within the germinal epithelium of control testis. (**b)** In the gc*Pex13*^*∆ex2/∆ex2*^*/*Stra8*-Cre*^+/−^ mice, the immunoreactivity was also extended focally up to the lumen of the seminiferous tubules. (**c)** In control, the azo-dye Evans Blue was clearly located in the suprabasal epithelium and in the interstitium. (**d)** In gc*Pex13*^*∆ex2/∆ex2*^*/*Stra8*-Cre*^+/−^ testes, Evans Blue was also dispersed adluminal of the germinal epithelium. Evans Blue is indicated in red. Nuclei DAPI stains are shown in grey. Bars = 50 µm.

**Figure 10:**
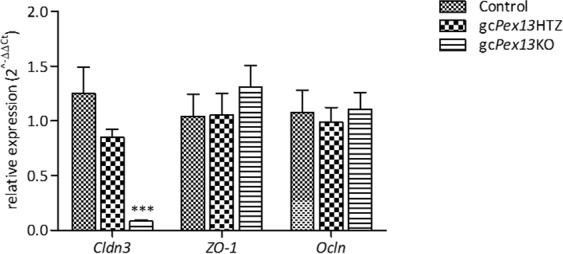
Relative gene expression of tight junction proteins in control group (square box bar), *Pex13*^*WT/*∆*ex2*^*/*Stra8*-Cre*^+/−^ (gc*Pex13*HTZ; brick bars) and gc*Pex13*^*∆ex2/∆ex2*^*/*Stra8*-Cre*^+/−^ mice (gc*Pex13*KO; horizontal line bars). QRT-PCR analysis was used to assess gene expression of occludin (*Ocln*), tight junction protein 1 (*Tjp1*) and claudin-3 (*Cldn3*). Data represent 3 independent experiments, each assayed in duplicate, representing gene expression of 3 individuals per genotype. Relative expression values were calculated by the ΔΔ*C*_*T*_-method. The standard error of the mean (SEM) is shown. Statistical significance was determined using t-test (Welch´s correction). *p ≤ 0.05; **p 0.001 < p ≤ 0.01; ***p ≤ 0.001.

In the double-immuno-fluorescent staining with anti-Vimentin, vimentin labelling displayed a characteristic pattern in control testes with Sertoli cell intermediate filaments extending from the basal compartment to the lumen, and lateral extensions (Fig. 8a,c,g,i). However, in *Pex13-*deficient mice, the immunostaining displayed an irregular pattern with intermediate filaments extending from the basal compartment to the lumen and lateral extensions that appear to enclose all germ cells, including luminal residing MNCs (Fig. 8d,f,j,l). As adjacent Sertoli cells form a barrier via tight junctions, irregularities in the cellular architecture of Sertoli cells in gc*Pex13*^*∆ex2/∆ex2*^*/*Stra8*-Cre*^+/−^ mice suggested pathological alterations in the blood-testis barrier (BTB). First, the distribution of the tight junction protein Claudin-11 was compared in tissue sections from control and gc*Pex13*^*∆ex2/∆ex2*^*/*Stra8*-Cre*^+/−^ (P60) testes. In control mice, the immunoreactivity of anti-Claudin-11 antibody was concentrated in the suprabasal layer of the germinal epithelium (Fig. 9a). However, in mouse testes with a *Pex13* deficiency, the immunoreactivity for anti-Claudin-11 was extended lateral and adluminal of the seminiferous tubules, suggesting an effect on the organization of the tight junction proteins (Fig. 9b). Consequently, the expression of genes encoding for several tight junction proteins that compose the BTB, including claudin-3 (*Cldn3*), zonula occludens 1 (*ZO1*) and occludin (*Ocln*), were quantified by qRT-PCR. The mRNA levels for *Ocln* and tight junction protein 1 (*Tjp1*) were comparable in all genotypes with only slight but no significant deviations in gc*Pex13*^*∆ex2/∆ex2*^*/*Stra8*-Cre*^+/−^ testes. However, the mRNA level of *Cldn3* was significantly (p > 0.0001) down-regulated in the gc*Pex13*^*∆ex2/∆ex2*^*/*Stra8*-Cre*^+/−^ testes compared to control testes (Fig. 10).

To functionally assess the integrity of the BTB, mice were injected *i*.*v*. with Evans Blue, an azo dye that binds to serum albumin. Because serum albumin cannot cross the functional BTB barrier, the adluminal compartment of seminiferous tubules would remain unstained. In healthy seminiferous tubules, Evans Blue was clearly located around spermatogonia, peritubular myoid cells and in interstitial Leydig cells (Fig. 9c). In gc*Pex13*^*∆ex2/∆ex2*^*/*Stra8*-Cre*^+/−^ mice, the distribution of injected Evans Blue was only slightly different. Compared to control animals, Evans Blue was partially dislocated to the adluminal compartment and showed a more irregular pattern (Fig. 9d).

### Accumulation of lipid droplets in gc*Pex13*^∆ex2/∆ex2^/Stra8-Cre^+/−^ testes

The Sertoli cells play a central role in maintaining the homeostasis level of cholesterol and lipid storage that are both required for spermatogenesis in terms of membrane remodeling of developing germ cells^38^.

**Figure 11:**
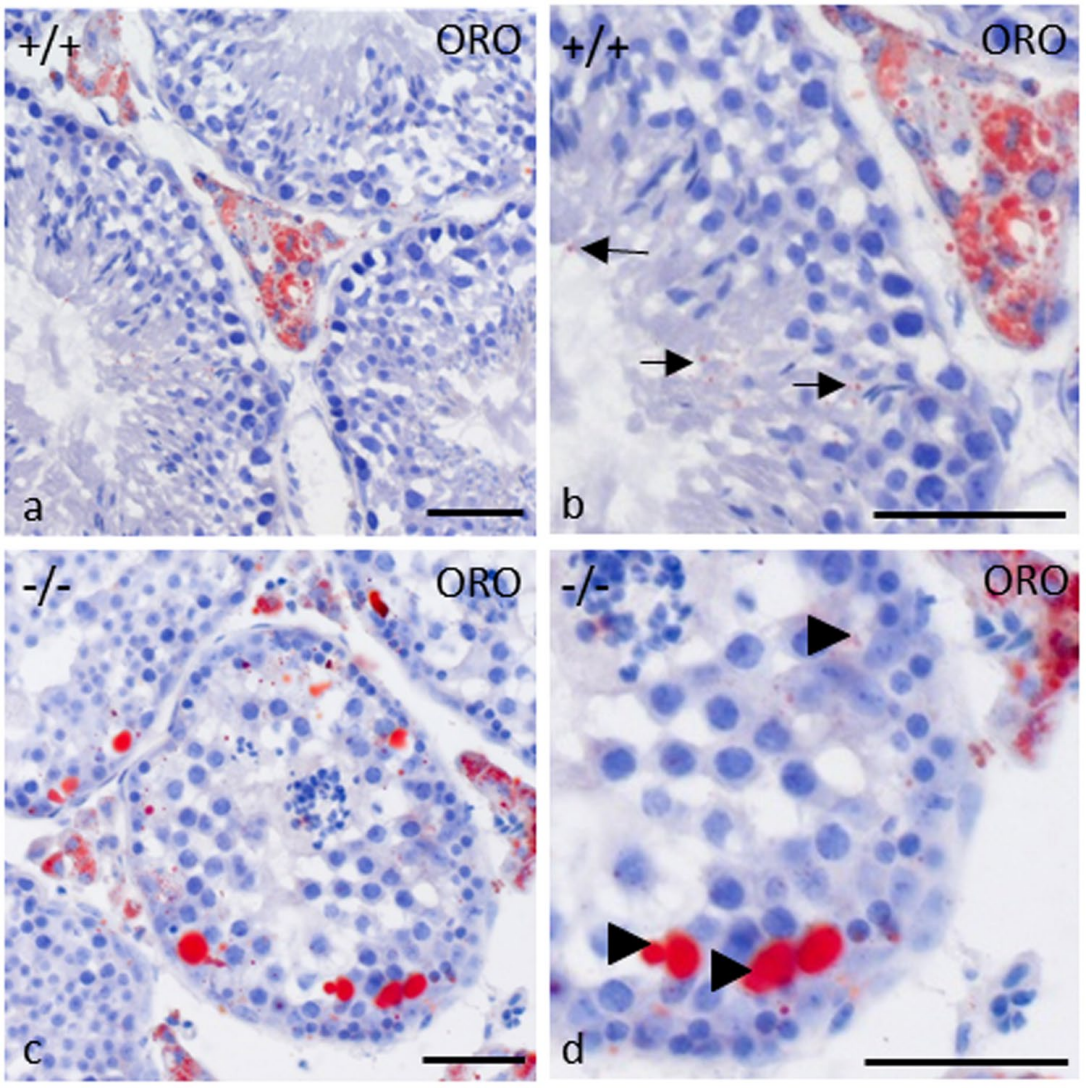
Histological detection of lipid droplets in control (+/+) and gc*Pex13*^*∆ex2/∆ex2*^*/*Stra8*-Cre*^+/−^ (−/−) testes by Oil Red O staining. (**a)** Lipid droplets were detected in Leydig cells and in (**b)** Sertoli cells (indicated by *arrows*) of control testes. (**c)** In gc*Pex13*^*∆ex2/∆ex2*^*/*Stra8*-Cre*^+/−^ testes, large inclusions of lipid droplets were found in the interstitial Leydig cells and in (**d)** Sertoli cells (indicated by *arrowheads*). (**b**,**d)** show higher magnification of testicular cross-sections of appropriate genotypes. Bars = 50 µm.

To test for triglycerides and cholesteryl esters, cryo-sections from control and gc*Pex13*^*∆ex2/∆ex2*^*/*Stra8*-Cre*^+/−^ mouse testes were analyzed by an Oil Red O (ORO) staining. In control testes, lipids were mainly assigned to Leydig cells (Fig. 11a,b). At stage VI of the germinal epithelium, small deposits of lipid droplets were present apically of Sertoli cells in control testes (Fig. 11b). In the gc*Pex13*^*∆ex2/∆ex2*^*/*Stra8*-Cre*^+/−^ testes, enlarged lipid droplets were accumulated in the basal part of Sertoli cells of stage IX to XI tubules (Fig. 11d), but were also detectable in the apical compartment of the germinal epithelium (Fig. 11c).

### Different concentrations of fatty acids in the gc*Pex13*^∆ex2/∆ex2^/Stra8-Cre^+/−^ testes

Gene expression studies on *Pex13*-deficient germ cells already indicated a disturbance in the metabolism of fatty acids as shown by differentially expressed genes of the peroxisomal *β*-oxidation pathway (Table 1). Thus, a total fatty acid analysis was done on whole testes biopsies by gas chromatography (Table 2).

**Table 2:** Total fatty acid composition up to C24, analyzed by gas chromatography of control and gc*Pex13*^*∆ex2/∆ex2*^*/*Stra8*-Cre*^+/−^ (gc*Pex13*KO) testes.

Function	Control	gc*Pex13*KO			*p*-Value
C14:0	5.19 ± 0.21	6.55 ± 0.22			0.0000074
C16:0	28.82 ± 1.16	23.11 ± 0.32			0.0000054
C18:0	9.11 ± 0.45	11.32 ± 1.01			0.0020610
C20:0	0.12 ± 0.01	0.19 ± 0.04			0.0031503
C22:0	0.10 ± 0.01	0.15 ± 0.02			0.0003257
C14:1n-5	0.03 ± 0.00	0.03 ± 0.00			0.6410983
C16:1n-7	1.09 ± 0.68	1.76 ± 0.80			0.1902945
C18:1n-7	3.14 ± 0.10	4.12 ± 0.17			0.0000037
C18:1n-9	10.30 ± 1.56	13.64 ± 1.44			0.0078814
C20:1n-9	0.34 ± 0.05	0.82 ± 0.11			0.0000207
C22:1n-9	0.05 ± 0.01	0.15 ± 0.06			0.0036308
C24:1n-9	0.27 ± 0.04	0.69 ± 0.20			0.0016675
C18:2n-6	4.25 ± 2.04	5.18 ± 1.84			0.4755331
C18:3n-6	0.06 ± 0.01	0.06 ± 0.00			0.6829483
C20:2n-6	0.23 ± 0.02	0.29 ± 0.06			0.0512692
C20:3n-6	1.48 ± 0.13	1.57 ± 0.18			0.4068332
C20:4n-6	11.38 ± 0.94	13.36 ± 1.57			0.0416817
C22:4n-6	1.34 ± 0.16	2.00 ± 0.35			0.0053801
C22:5n-6	12.57 ± 1.22	6.54 ± 0.72			0.0000124
C18:3n-3	0.55 ± 0.61	0.96 ± 0.68			0.3492519
C20:4n-3	0.02 ± 0.00	0.04 ± 0.01			0.0087583
C20:5n-3	0.27 ± 0.03	0.38 ± 0.03			0.0004958
C22:5n-3	0.55 ± 0.11	1.42 ± 0.19			0.0000194
C22:6n-3	8.08 ± 0.79	4.86 ± 0.52			0.0000634

SFAs and MUFAs were significantly increased in the gc*Pex13*^*∆ex2/∆ex2*^*/*Stra8*-Cre*^+/−^ testes, with the exception for C16:0 (palmitic acid). C22:6n-3 (DHA) and C22:5n-6 (DPA) were significantly decreased in the gc*Pex13*KO testes. Units are percentage of total ± SEM. Statistical significance was determined using t-test (non-parametric Mann-Whitney *U* test). The data of 5 biological replicates per group were considered and represented as a mean ± SD. *p ≤ 0.05; **p0.001 < p ≤ 0.01; *** p ≤ 0.001.

In Table 2, the relative tissue fatty acid pattern of control (n = 5) and gc*Pex13*^*∆ex2/∆ex2*^*/*Stra8*-Cre*^+/−^ testes (n = 5) is listed. The saturated FA levels were significantly increased in the gc*Pex13*^*∆ex2/∆ex2*^*/*Stra8*-Cre*^+/−^ testes, except for palmitic acid (C16:0). Stearic acid (C18:0) was elevated by 24.26%. The concentration of its derivative oleic acid (C18:1n-9) was elevated by 32.43% with 13.64 ± 1.44 compared to control testes (10.30 ± 1.56). In general, the n-7 and n-9 families were elevated in the gc*Pex13*^*∆ex2/∆ex2*^*/*Stra8*-Cre*^+/−^ testes, with a threefold increase of erucic acid (C22:1n-9).

Some n-3 PUFAs were significantly increased in the gc*Pex13*^*∆ex2/∆ex2*^*/*Stra8*-Cre*^+/−^ testes, including eicosatetraenoic acid (C20:4n-3) with a 100% increase, eicosapentaenoic acid (C20:5n-3) with a 40.74% increase and n-3 DPA (C22:5n-3) being threefold elevated. However, n-3 DHA (C22:6n-3) was significantly reduced with 4.86 ± 0.52 of total fatty acids, compared to control (8.08 ± 0.79). In the gc*Pex13*^*∆ex2/∆ex2*^*/*Stra8*-Cre*^+/−^, n-6 PUFAs, such as eicosatetraenoic acid (C20:4n-6) and docosatetraenoic acid (C22:4n-6) were increased.

The same was shown for triglycerides and phospholipids in whole testicular biopsies that contained less of n-6 docosapentaenoic acid (n-6 DPA, C22:5n-6) and n-3 docosahexaenoic acid (C22:6n-3), at least in phospholipids, as the measured values for n-3 DPA in triglycerides were below the measuring limit. Docosatetraenoic acid (C22:4n-6) and n-3 DPA (22:5n-3) were both increased in triglycerides and phospholipids.

**Figure 12:**
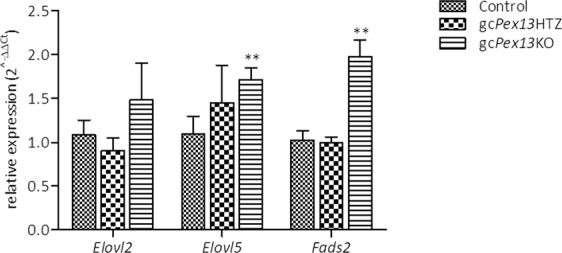
Expression profiles of elongases (*Elovl2*, *Elovl5*) and desaturases (*Fads2*) involved in PUFA synthesis in control (square box bars), *Pex13*^*WT/*∆*ex2*^*/*Stra8*-Cre*^+/−^ (gc*Pex13*HTZ; brick bars) and gc*Pex13*^*∆ex2/∆ex2*^*/*Stra8*-Cre*^+/−^ (gc*Pex13*KO; horizontal line bars) testes. Data represent 4 independent experiments, each assayed in duplicate, representing gene expression of 3 biological replicates per group. Relative expression values were calculated by the ΔΔ*C*_*T*_-method. The standard error of the mean (SEM) is shown. Statistical significance was determined using t-test (Welch´s correction). *p ≤ 0.05; **p 0.001 < p ≤ 0.01; ***p ≤ 0.001.

As the results of gas chromatography analyses revealed a decrease of n-3 DHA and n-6 DPA that are synthesized by elongases *Elovl 2* and *Elovl5* and desaturases, including *Fads1* and *Fads2*^39–41^, gene expression levels of these enzymes were further quantitatively assayed by qRT-PCR in RNA preparations of whole testes biopsies. Compared to control testes, *Fads2* expression in gc*Pex13*^*∆ex2/∆ex2*^*/*Stra8*-Cre*^+/−^ testes was significantly increased (p = 0.0032) (Fig. 12). The elongation of PUFA is controlled by ELOVL2 and ELOVL5. ELOVL2 is mainly involved in the elongation of C20 into C24:4n-6 and C22 into C24:5n-3 PUFAs, whereas ELOVL5 controls the elongation of C18 to C22. *Elovl2* expression was only slightly increased by trend in the gc*Pex13*^*∆ex2/∆ex2*^*/*Stra8*-Cre*^+/−^ testes compared to control mice. However, *Elovl5* showed significantly increased expression levels in the gc*Pex13*^*∆ex2/∆ex2*^*/*Stra8*-Cre*^+/−^ testes (p = 0.0031; Fig. 12) that is in accordance with the data obtained by gas chromatography measuring increased levels of eicosatetraonic acid (C20:4n-3), n-3 DPA (C22:5n-3) and adrenic acid (C22:4n-6) (Table 2).

## Discussion

The most severe forms of peroxisomal disorders are lethal, as lipids and cholesterol are a pre-requisite for cell structure. Moreover, ROS metabolism is essential to provide a toxic-free environment. In less severe forms of peroxisomal dysfunction, as described for patients with AMN and X-ALD, testicular alterations including degenerating Leydig cells, reduction of the seminiferous tubules or even spermatogenic arrest were diagnosed. In the present study, a conditional KO of the translocation machinery constituent *Pex13* was induced in pre-meiotic germ cells by using the *Cre*-*loxP* system, to analyze the effect of dysfunctional peroxisomes on spermatogenesis. We hypothesized severe disturbances on cellular level due to abolished peroxisomal function and provide an initial basic characterization of the phenotype.

Based on routine histological analyses, a different cellular composition in the germinal epithelium was observed in gc*Pex13*^*∆ex2/∆ex2*^*/*Stra8*-Cre*^+/−^ testes. Instead of round spermatids, MNCs were consistently present in all seminiferous tubules of juvenile and adult mice as consequence of truncated PEX13 (Fig. 3b). Male mice were sterile due to absent spermatozoa. During the course of regular spermatogenesis in mice, MNCs are frequently formed as consequence of apoptotic spermatogenic cells in the basal compartment that induce a defect in subsequent germ cell differentiation and thereby regulate apoptosis-inducing genes in higher differentiated germ cells^42^. In the gc*Pex13*^*∆ex2/∆ex2*^*/*Stra8*-Cre*^+/−^ testes, apoptotic spermatogonia and spermatocytes were present at higher rate (Fig. 5c,d). In addition, nuclei of MNCs became more condensed (Fig. 4f), as an early sign of apoptosis that was confirmed by antibody labelling with anti-BAX (Fig. 5d) and anti-Cleaved Caspase-3 (Fig. 5c). Apoptosis can be triggered by environmental factors^43^ or intrinsic factors leading to oxidative cell stress^44^. In the gc*Pex13*^*∆ex2/∆ex2*^*/*Stra8*-Cre*^+/−^ mice, the ROS degrading enzyme catalase was down-regulated and ROS scavenging PUFAs, such as n-6 DPA (C22:5n-6) and n-3 DHA (C22:6n-3) were significantly decreased (Table 2). Moreover, immunostaining against catalase showed a diffuse pattern in the cytoplasm of *Pex13*-lacking germ cells.

According to Santos *et al*., tissues from Zellweger Spectrum patients contained peroxisomal membrane structures, known as ghosts that lacked peroxisomal matrix proteins^45^. They report, that some peroxisomal proteins were synthesized normally in Zellweger syndrome but not assembled into peroxisomes. Whereas some proteins were rapidly degraded and thus not detectable in Zellweger cells, some peroxisomal enzymes, as e.g. catalase^46^, were located free in the cytoplasm^45^. This observation is in concordance to the peroxisomal protein pattern shown in our immunofluorescent analyses. In the gc*Pex13*^*∆ex2/∆ex2*^*/*Stra8*-Cre*^+/−^ testes, immunostaining not only against catalase but also peroxisomal 3-ketoacyl CoA thiolase, an enzyme that is involved in the degradation of straight chain acyl-CoAs (including the CoA esters of dicarboxylic fatty acids and eicosanoids), did not show a typical punctuate peroxisomal pattern in *Pex13*-deficient germ cells of gc*Pex13*^*∆ex2/∆ex2*^*/*Stra8*-Cre*^+/−^ mice (Fig. 8j–l), as a sign for a defect in peroxisomal matrix protein import. The effects of inactivated *Pex13* were already reported by Maxwell *et al*.^26^. Mutant *Pex13* mouse pups, exhibiting the Zellweger syndrome, lacked morphologically intact peroxisomes, displayed defective peroxisome biogenesis and showed deficient import of matrix proteins.

The elongation of FAs ≥ C16, that are either derived by food intake or *de novo*, occurs in the endoplasmic reticulum^47,48^. Fatty acid metabolism of short and medium chain length FAs takes place in mitochondria whereas peroxisomes metabolize VLCFA. These will then be transported into mitochondria for subsequent oxidation to CO_2_ and H_2_O^49^. The analyses of total fatty acids showed a reduction of n-6 DPA (C22:5n-6) and n-3 DHA (C22:6n-3) that can either be consumed by food intake or biosynthesized from corresponding precursor fatty acids, as 18:3n-3 and 18:2n-6^50^, of which the latter was slightly increased in the gc*Pex13*^*∆ex2/∆ex2*^*/*Stra8*-Cre*^+/−^ testes. N-6 docosatetraenoic acid (C22:4n-6) and n-3 DPA (C22:5n-3) were also increased in the gc*Pex13*^*∆ex2/∆ex2*^*/*Stra8*-Cre*^+/−^ testes. Interestingly, in a study from Petroni *et al*. (1998), increased formation of the intermediate n-3 DPA (C22:5n-3) was found in skin fibroblasts of Zellweger patients when analyzing the *β*-oxidation of arachidonic acid (C20:4n-6). Moreover, they could show an accumulation of adrenic acid (C22:4) in these samples by HPLC analyses. The authors conclude an impaired synthesis of DHA from EPA in the steps beyond the formation of the intermediates with carbon length C22 and C24. They assume that impaired peroxisomal *β*-oxidation, as characteristic of Zellweger patients, leads to a redirection of the conversion of arachidonic acid through elongation/desaturation pathway with consequent accumulation of the C22:4 product^51^.

The biosynthesis pathway of C22:5n-6 and C22:6n-3 requires sequential desaturation and elongation steps on the ER and final peroxisomal *β*-oxidation. Desaturation is controlled by *FADS1*, coding for ∆5-desaturase^52^, as well as *FADS2* that encodes for a ∆6- and ∆8-desaturase, and as recently demonstrated, is also involved in ∆4 desaturation of C22:6n-3 and C22:5n-6 biosynthesis^53^. In the gc*Pex13*^*∆ex2/∆ex2*^*/*Stra8*-Cre*^+/−^ mice, *Fads2* was significantly increased. In addition, reduced gene expression of *Acox1* that catalyzes straight-chain saturated and unsaturated VLCFAs during peroxisomal *β*-oxidation (including C22:6n-3)^54^ was measured in the gc*Pex13*^*∆ex2/∆ex2*^*/*Stra8*-Cre*^+/−^testes (Table 1). Interestingly, DHA synthesis from C18:3n-3 or C22:5n-3 was shown to be defective in Zellweger Syndrome^55^. The group of Su *et al*. (2001) even observed an accumulation of direct precursors of DHA, C24:5n-3 and C24:6n-3 in ACOX-deficient cell lines compared to control fibroblasts^55^.

Based on our results, we do not expect a problem of fatty acid transport into germ cells^56,57^ as the fatty acid levels are not equally altered. One putative conclusion could be that the different concentrations of fatty acids in the gc*Pex13*^*∆ex2/∆ex2*^*/*Stra8*-Cre*^+/−^ testes are due to altered degradation and to altered incorporation of fatty acids in e.g. triglycerides, phospholipids which needs to be tested in further studies. Taking all data together, we can state that fatty acids being elongated were increased, whereas fatty acids that are supposed to be oxidized via peroxisomal *β*-oxidation, were decreased which we assume might be associated with a defect of fatty acid import into the organelle as consequence of the *Pex13* KO.

However, one limitation of the present study is the lipidomic and mRNA analyses on whole testes biopsies and not on single cells: It does not allow a conclusion whether the changes in FA metabolism were the consequence of the different cellular composition of the testis (absence of spermatozoa and presence of MNCs) or if the physical membrane characteristics were altered, as already described for patients with a peroxisomal biogenesis disorder^58^. Different cell separation techniques were performed, including BSA gradient, cell cytometry, laser capture microdissection (LCM) and counterflow centrifugation elutriation (CCE) to gain single cell populations. However, all cell separation techniques were not reliable to purify MNCs from gc*Pex13*^*∆ex2/∆ex2*^*/*Stra8*-Cre*^+/−^ mice. They were still found in all samples and could only be enriched to a purification rate of 60%. Moreover, many cytoplasmic cells lacking nuclei were found in the sample, indicating either a disruption of MNCs during the procedure or even a high apoptotic rate during spermatogenesis.

A particular focus was placed on the polarization of Sertoli cells and tight junction proteins as their major function is protecting germ cells from the circulatory and lymphatic system and therefore providing them an immune-privileged microenvironment for meiosis^59,60^, by preventing trespassing of molecules larger than 1,000 Da. The BTB is assembled by specialized junctions between Sertoli cells, including basal ectoplasmic specialized (ES) protein complexes, gap junction protein complexes and actin-based tight junction protein complexes^61–63^. The main contributors to maintain the BTB integrity are claudin-3^64^, 5^65^ and −11^66^ as well as occludin^67^. Immunolabelling against claudin-11 already indicated a severe disturbance in the structural organization of the BTB in the gc*Pex13*^*∆ex2/∆ex2*^*/*Stra8*-Cre*^+/−^ mice (Fig. 9b). Moreover, *Cldn3* gene expression was significantly down-regulated, whereas *ZO1* and *Ocln* were slightly up-regulated (Fig. 10). Overall, the tight junction proteins were only differently expressed but not fully absent in the gc*Pex13*^*∆ex2/∆ex2*^*/*Stra8*-Cre*^+/−^ mice.

In X-ALD patients with mutated *ABCD1*, the endothelial tight junction proteins, e.g. CLDN5 and ZO-1, are likewise differentially regulated, leading to a disruption of the blood-brain barrier (BBB)^68–70^. The functional analysis based on Evans Blue injections revealed a diffusion of the dye into the adluminal compartment, surrounding some MNCs in the gc*Pex13*^*∆ex2/∆ex2*^*/*Stra8*-Cre*^+/−^ testes (Fig. 9d). These data indicated a disruption of Sertoli cell polarity, as the cytoskeletal marker vimentin showed an irregular pattern in the gc*Pex13*^*∆ex2/∆ex2*^*/*Stra8*-Cre*^+/−^ testes (Fig. 8f,l), displaying not only lateral but also cytoskeletal projections from the basal compartment to the lumen, whereby surrounding single germ cells, including MNCs, in the adluminal compartment of the germinal epithelium. However, the functional integrity of the BTB must be further assessed^71^.

To conclude, the present data gives evidence for a very complex mechanism to ensure spermatogenesis and to maintain the germinal epithelium in a structured organization. The defect in the peroxisomal compartment influenced the cellular architecture as well as cell differentiation within the germinal epithelium. Moreover, the *Pex13* KO caused alterations in the fatty acid synthesis and thus different levels of SFAs, MUFAs and PUFAs. However, we were not able to examine single mechanisms that could explain the present phenotype. It still remains unclear whether the failure in peroxisomal biogenesis in pre-meiotic germ cells had an effect on peroxisomal *β*-oxidation, leading to an imbalance of metabolites for mitochondria, or whether the organelle interplay was disturbed inducing defects in mRNA expression and thus alterations in fatty acid synthesis. Moreover, one limitation of the study was that all analyses were performed on whole testis biopsies. So far, no suitable approach was found to isolate, purify and enrich MNCs to analyze pathways and fatty acid synthesis in single cells.

## Supplementary information

Supplementary Table T1

## Data Availability

The datasets generated and analyzed during the current study are available from the corresponding author on reasonable request.
