# Supplementary material for: A defect in the peroxisomal biogenesis in germ cells induces a spermatogenic arrest at the round spermatid stage in mice
Source: Sci Rep. 2019 Jul 2;9:9553. doi: 10.1038/s41598-019-45991-6 (PMC6606614; doi:10.1038/s41598-019-45991-6)
Supplement: Supplementary file 1 — Supplementary Table T1 [file 41598_2019_45991_MOESM1_ESM.pdf]

**Supplementary material**

A defect in the peroxisomal biogenesis in germ cells induces a spermatogenic arrest at the round spermatid stage in mice

Ann-Kristin Brauns, Markus Heine, Klaus Tödter, Eveline Baumgart-Vogt, Georg H. Lüers, Udo Schumacher

**Supplementary Table T1**: Oligonucleotides

| Gene | Symbol | Accession no. | | Primer sequence | Ann. Temp. (°C) | Length (bp) |
|---|---|---|---|---|---|---|
| ATP-binding cassette, sub-family D (ALD), member 1 | *Abcd1* | MGI:1349215 | For<br>Rev | GCCAGCCTCAACATCAGG<br>ACTCTTGCCACAGCCATTG | 58.1<br>58.4 | 72 |
| Acyl-Coenzyme A oxidase 1, palmitoyl | *Acox1* | MGI:1330812 | For<br>Rev | TGGAGATCACGGGCACTTAT<br>TTCCAAGCCTCGAAGATGAG | 58.5<br>57.3 | 65 |
| Acrosomal vesicle protein 1 | *Acrv1* | MGI:104590 | For<br>Rev | TGGAGAAGGAGTATGCACCA<br>ACCCTTGAACCATGAACTGG | 58.1<br>57.7 | 92 |
| Actin beta | *Actb* | MGI:87904 | For<br>Rev | GGATGCAGAAGGAGATTACTGC<br>CCACCGATCCACACAGAGTA | 58.9<br>58.8 | 94 |
| Catalase | *Cat* | MGI:88271 | For<br>Rev | CCTTCAAGTTGGTTAATGCAGA<br>CAAGTTTTTGATGCCCTGGT | 56.9<br>56.8 | 80 |
| Claudin 3 | *Cldn3* | MGI:1329044 | For<br>Rev | GTACAAGACGAGACGGCCAA<br>CGTACAACCCAGCTCCCATC | 59.4<br>61.4 | 175 |
| ELOVL fatty acid elongase 2 | *Elovl2* | MGI:1858960 | For<br>Rev | GAGAAGGTGATGTCCGGGTAG<br>ACATGGACGCGTGGTGATAG | 61.8<br>59.4 | 144 |
| ELOVL fatty acid elongase 5 | *Elovl5* | MGI:1916051 | For<br>Rev | TTCCTCTTGCATCGCGGCT<br>CCATCCTTTGACTCTTGTATCTCGG | 58.8<br>63.0 | 200 |
| Fatty acid desaturase 2 | *Fads2* | MGI:1930079 | For<br>Rev | ACCTTCCGTTGGGAGGAGAT<br>GGAAGGCATCCGTAGCATCTT | 59.4<br>59.8 | 160 |
| Glyceraldehyde-3-phosphate dehydrogenase | *Gapdh* | MGI:95640 | For<br>Rev | AGCTTGTCATCAACGGGAAG<br>TTTGATGTTAGTGGGGTCTCG | 58.2<br>57.7 | 62 |
| Glyceronephosphate O-acyltransferase | *Gnpat* | MGI:1343460 | For<br>Rev | GCAGCTTTCGTGAGACTGG<br>GCAGGCCCGTTCACATAATA | 58.8<br>58.0 | 77 |
| Hydroxysteroid (17-beta) dehydrogenase 4 | *Hsd17b4* | MGI:105089 | For<br>Rev | AGCATGGGACCATATGAAGAA<br>TATAATTCGCCTGGCCAAAG | 56.8<br>55.9 | 95 |
| Occludin | *Ocln* | MGI:106183 | For<br>Rev | GTGAGCACCTTGGGATTCCG<br>GGGTTTGAATTCATCAGGTCTGT | 61.4<br>58.9 | 153 |
| Peroxisomal biogenesis factor 13 | *Pex13* | MGI:1919379 | For<br>Rev | TAGACCAGACTGGCTTCGAACT<br>CATGCCTTTAATCCCAGCAC | 60.8<br>57.1 | 60 |
| Floxed peroxisome biogenesis factor 13 | *Pex13loxP* | MGI:919379 | For<br>Rev | ATGGCTCCCAAGTTAGTTCTG<br>TCTGTTTCCCTCCCACCTC | 57.9<br>58.8 | 490 (WT)<br>540 (loxP) |
| Deleted exon 2 of Pex13 | *Pex13Δ* | MGI:919379 | For<br>Rev | TGGCTCCCAAGTTAGTTCTGTC<br>CCTCTCTATTTGTTGCTTACCCC | 60.3<br>60.6 | 410 |
| Peroxisomal biogenesis factor 14 | *Pex14* | MGI:1927868 | For<br>Rev | ACTCCGCAGCCATACAGC<br>CTGCCATGATGATAGCCAAG | 59.8<br>56.3 | 70 |
| Peroxisomal biogenesis factor 19 | *Pex19* | MGI:1334458 | For<br>Rev | TGCTGTACCCATCCCTGAA<br>GGAGGAGTGGAGTCCTGGT | 57.9<br>59.9 | 76 |
| Protamine 1 | *Prm1* | MGI:97765 | For<br>Rev | CACAGCCCACAAAATTCCA<br>CAGAGCAGGGGACACCAC | 56.3<br>59.6 | 67 |
| Synaptonemal complex protein 3 | *Sycp3* | MGI:109542 | For<br>Rev | GGGACAGCGACAGCTCAC<br>CCAGATTTCCCAGAATGCTT | 60.4<br>55.7 | 82 |
| Superoxide dismutase 1 | *Sod1* | MGI:98351 | For<br>Rev | CAGGACCTCATTTTAATCCTCAC<br>TGCCCAGGTCTCCAACAT | 56.9<br>58.1 | 78 |
| Stimulated by retinoic acid 8 | *Stra8* | MGI:107917 | For<br>Rev | AGATGCCAGGACATCAGGAACCTG<br>ATCAGCCACACCAGACACAGAGATC | 64.4<br>64.6 | 236 |
| Tight junction protein 1 | *Tjp1* | MGI:98759 | For<br>Rev | GGAGATGTTTATGCGGACGG<br>CCATTGCTGTGCTCTTAGCG | 59.4<br>59.4 | 136 |
